# Association of American Physicians

**Published:** 1888-10

**Authors:** 


ASSOCIATION OF AMERICAN PHYSICIANS.

Professor W. H. Draper, of New York, delivered the President's address, and offered some reflections upon the relation which scientific and practical medicine bear to each other.

He said: We may differentiate the science from the art of medicine, but we cannot practically dissociate them. In their ideal union they are interwoven like warp and woof, built into each other like foundation and superstructure. Scientific medicine, to be sure, is not always practical, but it is ever striving to become so; and practical medicine, though not always in a strict sense scientific, is constantly tending to that end. In medicine, perhaps less than in any other department of human activity, has the distinction between the art and the science been sharply drawn. In spite of this close mutual relation, the worker in the science and the worker in the art occupy essentially distinct positions, and the aim and the methods peculiar to each must be constantly kept in view. The one is a naturalist, the other an artsman. The tendency is to accentuate the distinction. The obvious results of this specializing it is hardly necessary to point out. The observations of the physician have not only suggested to the scientist some of the most interesting problems in biology but some of the most important factors in their solution.

Dr. W. W. Johnston, of Washington, read a paper entitled, "Geographical Distribution of Typhoid Fever in the United States."

The paper was based largely upon the answers returned from three hundred and fifty physicians in various parts of the country, and colored maps showing the distribution of typhoid, and of malarial fever were exhibited. Dr. Johnston's conclusions are: 1. That typhoid fever has a much wider distribution in this country than is accepted by the majority of medical men, and that its prevalence is very great in all of the regions reviewed in the paper. 2. That the varieties and modifications of type are very numerous, covering a wide range, and simulating types of malarial disease. 3. That the prevailing type is without many, sometimes without any, of the more characteristic symptoms of the disease. 4. That the principal direction in which clinical study can be useful in the present stage of our knowledge is in the collection of cases of undoubted typhoid fever; cases in which the diagnosis is made certain by characteristic symptoms, especially by post-mortem examination, in which there have been phenomena supposed to show malarial infection. 5. By the study of the localities of occurrence of such cases, by the finding of them, perhaps, in non-malarial regions, by the study of the blood and stool micro-organisms. 6. That no true progress can be made until the true value of the malaria-like symptoms are determined.

Dr. JAMES H. HUTCHINSON, of Philadelphia, read a paper on "The Management of the Stage of Convalescence in Typhoid Fever."

With the return of the normal temperature, the physician is prone to overlook the danger. The authorities differ greatly as to the rules laid down for the management of this stage, some advising the administration of solid food so early as the second day after the cessation of the fever; others not until a week or ten days have passed. Dr. Hutchinson favors the continued use of the milk diet, with a little milk-toast, and at the end of two weeks butcher's meat. The only objection to be urged against the milk diet are the wishes of the patient and the tendency it occasionally has to produce constipation.

The administration of alcohol may be withheld in many instances; but, on the other hand, it may, for the first time, become necessary, and no doubt convalescence is often very much hastened by its use.

Dr. PEABODY, of New York, in opening the discussion, stated that he did not believe that errors in diet were so frequently the cause of recrudescence as was formerly supposed. In regard to ulcers as a cause of diarrhœa, he is of the opinion that their presence has nothing whatever to do with its production, a conclusion derived from the frequent study of cases at the autopsy table.

Dr. ORD, of London, in a general way, agreed with the reader of the paper, but thought that one cannot lay down hard and fast rules. One must be guided, in a measure, by the desires of the patient, and must give in to these desires. He referred to a hospital case which ran the usual course up to a certain period in the convalescence, when there was added a more extraordinary range of temperature for days, with delirium, and an abiding desire for food. The patient had been allowed milk and beef-tea, and for ten days two eggs daily. She was given boiled sole, and within twenty-four hours her temperature was normal, and convalescence was thereafter uninterrupted. We should study the individual as well as the fever.

Dr. KINNICUTT, of New York, remarked that he considered relapse to be very often a result of indiscretion in diet, and that therefore the old way of adhering strictly to a liquid diet was the best.

Dr. GEORGE ROSS, of Montreal, then presented a communication entitled, "Some Forms of Paralysis after Typhoid Fever."

It is not to be wondered at that the nervous system suffers after typhoid. The signs of exhaustion of the nervous system are constant, and generally in proportion to the severity of the fever. The nervous disturbance may be general, or some part of the spinal cord or some one or more of the spinal nerves exhibit altered functions. The nervous phenomena almost invariably are both motor and sensory; always in the case of spinal nerves, never in any of mixed. According to Nothnagel, the order of frequency of these affections is as follows: 1. Parts by one nerve, as the ulnar or peroneal. 2. Paraplegia, preferably of the lower extremities. 3. Less frequently, one extremity, either upper or lower, or two extremities in crossed order.

4. Simple alteration of sensibility. The history of a case of paraplegia resulting directly from typhoid fever, and ending in recovery, was given in detail, and also the history of a case which presented paralysis involving all the limbs and the muscles of the palate.

Dr. FREDERICK FORCHEIMER, of Cincinnati, read a paper on "Fatty Heart."

The paper was based on a collection of one hundred and twenty-two cases—eighty-eight males and thirty-four females—fifty-nine of whom were below sixty years of age and forty above. The relation of obesity to fatty heart was considered, and the difficulties attending the diagnosis of the affection dwelt upon.

Dr. JAMES C. WILSON, of Philadelphia, presented a communication entitled, "Causal Therapeutics in Infectious Diseases."

The paper was based upon an experimental study of the effect of hypodermic injections of calomel in the treatment of five cases of typhoid fever, all severe, and three of pulmonary tuberculosis. In all there was a distinct amelioration of symptoms, and a modification of the temperatures. The drug was given at intervals of four or five days.

### SECOND DAY.

Dr. ROBERT T. EDES, of Washington, read a paper on "The Absolute and Relative Value of Albumen and Casts, and of Renal Inadequacy, in the Diagnosis and Prognosis of Diseases of the Kidney."

The term albuminuria has held a place in medical nomenclature, until comparatively recently, synonymous with Bright's disease. The writer referred to recent and most extensive observations on albuminuria. The varieties of albumen were then considered. Dr. Munn's observations were cited to show that many cases of intermittent albuminuria, when followed out carefully, are found to have developed Bright's disease. Four of Dr. Munn's sixty-nine cases died within three years. Many such are met with in business men of middle-life, and of over-weight. The great diagnostic significance of casts, and their variety, was next touched upon. A question of great interest is, whether we can detect a pathological condition of the kidney in the diminution of any of the constituents of the urine. Dr. Noyes' paper in the *American Journal of the Medical Sciences* is of interest in this connection. Long after the presence of kidney disease is established, the daily quantity of urea excreted may be far above the physiological limit. A very badly damaged kidney may, in fact, get rid of a large quantity of urea. Cases in illustration of the fact that the excretion of urea and other solids does not necessarily fall below the physiological limit. The question of food is here of the greatest importance.

Professor JAMES TYSON, of Philadelphia, presented a communication entitled, "The Relation of Albuminuria to Life Insurance."

No system of life insurance is perfect which does not include those who are apparently healthy and those who are not. Certain applicants presenting themselves for examination for life insurance are wrongfully rejected, because of the presence of albumen in the urine. After this introduction the writer went on to mention the different terms used to indicate the particular forms of albuminuria under consideration, and gave his decided preference to the term "functional." He stated conditions which, if observed by competent and well-trained observers, if it were always possible to get such, would enable the companies to save these risks. These are: 1. The applicant must in all other respects present the signs of good health. 2. The albuminuria must be unaccompanied by tube-casts, however perfect may be the health in other respects; albumen and tube-casts conjoined always meaning structural changes. 3. The specific gravity of the urine, that is, the "real" specific gravity (that of the quantity for the twenty-four hours), should not be lower than 1.015–1.025. Great care must be taken to secure the "real" specific gravity, as it would be unfair, to reject the candidate on account of the specific gravity of a single specimen. 4. The signs of hypertrophy of the left ventricle, and the existence of high vascular tension associated with albumen, would exclude the candidate. 5. The age of the applicant is a highly important consideration. It is doubtful whether any person forty years of age with functional albuminuria should be accepted, unless at least he has been long under the observation of a competent and conscientious examiner. 6. The presence of true gout in any case should decide against the person, because gout is always, sooner or later, followed by interstitial nephritis. Finally, retinal changes, such as are associated with nephritis, should exclude the applicant. In conclusion, the writer does not claim, of course, that we are in a position to put these conditions in operation, but believes, as we are enabled gradually to secure the desired education and training in medical examiners, the applications of these conditions will be possible. The absence of the albumen from the urine passed on rising in the morning is an important aid in the diagnosis of functional albuminuria, but not an essential one.

Professor A. L. LOOMIS, of New York, read a paper entitled, "The Cardiac Changes in Chronic Bright's Disease."

Pathological processes become physiological in that they tend to the preservation of health and the prolongation of life. We have two widely different conditions tending to produce arterial tension—conditions which may act singly or conjointly—and affording a rational explanation of the varying amount of cardiac hypertrophy found in apparently similar renal conditions. First, a general and primary arterial sclerosis, to which both the cardiac and renal changes are secondary; and, second, arterial as well as cardiac hypertrophy, which are compensatory and conservative, as is cardiac hypertrophy in aortic lesions.

Professor SAMUEL C. CHEW, of Baltimore, Maryland, followed with a paper on "The Relation Between Chronic Interstitial Nephritis and Angina Pectoris."

After stating that there was no systematic account of the relations between the diseases to be found in any of the writers on the subject, he proceeded to consider the question chiefly from a clinical point of view.

Professor J. M. DACOSTA, of Philadelphia, presented a paper on the "Treatment on Valvular Affections of the Heart."

He began with a reference to the prevailing ideas on the treatment of these affections. The presence of a valvular affection is not the key-note to be taken. The indications on which the true treatment should be based are: 1, The state of the heart-muscle and heart-cavities; 2, the rythm of the heart; 3, the condition of the arteries and veins of the body; 4, probable length of disease; 5, the general health of the patient; 6, the secondary results of cardiac affection.

THIRD DAY.

Dr. EDWARD C. SEGUIN, of New York, presented a paper on "The Relation Between Trophic Lesions and Diseases of the Nervous System."

The author excluded from consideration vaso-motor disorders, and diffused or quantitative nutritional changes, partly because he considers them to be a distinct subject, and partly because their study would be beyond the scope of a short paper.

He divided the so-called "trophic lesions" into two great classes. In one the lesions are probably mostly due to the action of extraneous causes, and are preventable. The second class embraces lesions which seem to be directly due to the nervous disease, and which are its necessary and unpreventable results.

He endeavored to show that lesions of the second class, which alone merit the name of *trophic lesions*, occur in organs and tissues which are anatomically continuous, and whose life or preservation of structure and function is associated or interdependent.

He concludes that disease of the nervous system produces true trophic lesions when it interferes with the associated or interdependent life of continuous tissues. This proposition he offered only as a partial and preliminary answer to the question under discussion.

Professor HORATIO C. WOOD, of Philadelphia, followed with a paper on the same subject. His propositions and conclusions were as follows: 1. It is physiologically proven that the nervous system directly affects general nutrition. 2. Various lesions are the immediate result of previous nerve disease. 3. In various cases the lesions are not preceded by circulatory disturbance. 4. No known vaso-motor condition is capable of causing many of these lesions. 5. Therefore it is absurd to attribute changes to preceding vaso-motor changes.

Dr. ORD, of London, opened the discussion by stating these so-called trophic disturbances might be produced in three ways: 1, Through disease of the spinal cord; 2, through disease of nerves; 3, in a reflex way. In illustration, he mentioned a case of chronic hypertrophic cervical pachymeningitis in which there was a wasting of the tissue in the digits of the affected arm, and osteo-arthritis; also the case of a man who, through violent exercise, had lost power in his arms, and whose muscles underwent extreme wasting. There was also wasting of the skin. In conclusion, Dr. Ord spoke of the swelling of the joints of women approaching the climacteric. Various other reflex disturbances were referred to.

Dr. HENRY P. BOWDITCH, of Boston, considered that much might be gained by studying the subject on simpler lines; that therefore it would be well to limit the question to a consideration of its relation to muscles, for in muscles we have not two sets of nerves anatomically distinct, at least such are not demonstrable; we have not the "anabolic" and "katabolic" or "metabolic" nerves. It were better to confine the term "trophic" to nitrogeneous metabolism of muscle.

Dr. FERRIER, of London, said that the nervous system has a direct influence on the nutrition of tissue altogether apart from vaso-motor influence. The influence of nerves is amply demonstrated in the case of muscles; whether it be in case of the skin is not so certain. Possibly there are centres which preside over nutrition. It is an interesting question whether there are "trophic" nerves apart from motor and sensory.

Mr. VICTOR HORSLEY, of London, called the attention of his hearers to the recent and interesting investigations of his friend, Dr. Frederick Mott. To Dr. Mott belongs the credit of being the first to show that atrophy is a true active dystrophy and not a mere passive change. By cutting of the cauda equina he demonstrated an atrophy of the femur. The whole of its inner surface was lined with osteoclasts.

Dr. FRANCIS T. MILES, of Baltimore, next read a paper on "The Effect of Concussion of the Spine on the Reflexes.

Dr. W. H. WELCH reported the results of experiments relating to the thyroid gland of the dog performed by Dr. W. S. Halsted in the Pathological Laboratory of the Johns Hopkins University. Extirpation of both lobes of the gland of the dog was found to be uniformly fatal in from two days to three weeks, with symptoms similar to those noted by previous experimenters. A large number of experiments were performed to test the remarkable statement of Munk that these symptoms and the fatal result are not due to the loss of the thyroid gland, but are referable to some undefined injuries attending the operation of removal. Munk found that eight dogs survived the isolation of the gland by ligation of all the arteries and nerves entering it, provided the wound healed by first intention. In the experiments reported by Dr. Welch, with strict antiseptic precautions, the two lobes of the gland were isolated and all their vascular and nervous connections ligated. Under these circumstances the gland underwent coagulation-necrosis, and although in eight cases primary union of the wound was obtained, the animals died with the usual symptoms of extirpation of the entire gland. These results, therefore, are directly opposed to those reported by Munk.

Dr. T. Mitchell Prudden exhibited a set of microscopic specimens of myxœdema.

Dr. E. O. Shakespeare, of Philadelphia, exhibited photo-micrographs of various micro-organisms.

Dr. Jacobi, of New York, read a paper on "The Pathology of the Thymus Gland."

Because of the great length of the paper only a few extracts are given. The literature of the thymus for the last ten or twenty years treats more of its histology and embryology than of its pathology. The author referred to the fact that there is no certain size of the thymus; that, indeed, a great many different sizes and weights have been taken to be normal by different writers. Dr. Jacobi referred to the very doubtful connection between an alleged hypertrophy of the thymus and a so-called laryngismus stridulus. Still he admitted there are a few cases in which the sudden death of infants could not be explained any better than by the hypertrophy of the gland. Briefly he alluded to inflammations and suppurations of the gland, and to the occurrence of punctated or larger hæmorrhage into the tissue, and to the occurrence of malignant growths in the organ. Among them he mentioned endothelioma, lymphoma, fibroma, sarcoma, carcinoma, and myxoma, which have been described; the majority of them, however, are decidedly doubtful, particularly because it can be proven that a number of these pseudo-plasms did not originate in the thymus, but in the thyroid gland, or in a neighboring lymphatic gland. For the last eight months he had given his particular attention to the study of the thymus, with regard to tuberculosis, syphilis, and diphtheria. The material for the microscopical studies has been gathered from different institutions in New York City. These microscopical examinations were made by Dr. Henry Koplik, in the Laboratory of the College of Physicians and Surgeons. A few of the conclusions arrived at in regard to tuberculosis are as follows:

In cases of tuberculosis of the thymus here presented, tubercle tissue appeared in the following forms:

1. As miliary tubercles, composed entirely of small, round, or polygonal cells, with a reticulum basement substance in the recent state. In the later stages these miliary tubercles or granula may, at their centres, undergo cheesy metamorphosis (coagulation necrosis).

2. Miliary granula are also found which show, in their centres, the presence of giant cells.

3. Large, cheesy areas, in the periphery of which we still find miliary tubercles, of granula, composed of giant cells, around which are arranged spheroidal or polygonal cells in a fine reticulated basement substance.

4. In all cases the arteries of the adjacent areas of the thymus tissue were the seat of a typical endarteritis (in some cases obliterating) of a tubercular character.

5. A very careful and painstaking examination of the above forms showed tuberculosis present in all cases. In most of the above forms we had also the presence of the bacillus in the walls of the arteries and arterioles undergoing tubercular changes, and the lumen of the vessels was the seat of obliterating changes.

6. In the thymus, tuberculosis may appear simply as so-called tubercle tissue, an infiltration of the tissue of the gland or organ with spheroidal or polygonal cells, held together by a delicate basement substance ; this tissue has no characteristic arrangement ; the arteries in such areas may be the seat of obliterating processes. In all of the cases examined by us there were present the characteristic bacillus tuberculosis.

Syphilis results in the thymus in two changes, both in the accumulation of hyperplastic connective-tissue, and, as in one case, in the formation of a gumma. Diphtheria results in general parenchymatous degeneration.

Finally, persistent thymus, found in a man, aged twenty-six, was made the subject of extensive studies, and it was discovered that retrograde metamorphosis was in full progress ; the parenchyma being in a condition of fatty degeneration, and the stroma being replaced almost entirely by fat tissue.

The time allotted to the paper was mostly taken up by the demonstration of frozen sections illustrating the normal topography of the thymus ; also of photographs of the same, and of microscopical drawings of the pathological changes.

Mr. F. S. Eve, of London, made some remarks on the work of Mr. A. Lingard and himself on "Syphilis," an account of which work was published in *The Lancet* of April, 1886. The bacillus found during their investigations they do not claim to be the specific germ of the disease. Mr. Eve also spoke on the tubercular origin of lupus, and referred to studies of his own in this direction. In one instance he succeeded in producing lupus in a rabbit by inocculation of tissue from a case of the disease.

Dr. William T. Councilman, of Baltimore, presented several microscopic and macroscopic specimens illustrating bovine tuberculosis.

Dr. George M. Sternberg, U. S. A., owing to the lateness of the hour, did not read his paper on "Recent Researches Relating to the Etiology of Yellow Fever," but described the method followed in these researches, which were carried on at Havana, and stated that he had not arrived at any definite result as to the specific etiological element of yellow fever. He had made many cultures from the blood of persons just dead of this disease, as well as from the liver, kidneys, and urine. The results were negative as regards those made from the blood and urine. From the kidneys and liver numerous organisms were obtained, but in no instance did he obtain the micrococcus of Freire. Various tube cultures were shown.

